# Intraoperative bacterial cultures fail to reliably predict the bacterial spectrum encountered during infectious complications after appendicitis

**DOI:** 10.1007/s13304-023-01698-y

**Published:** 2023-12-07

**Authors:** Jens K. H. Strohäker, Martin J. Brüschke, Robert Bachmann, André L. Mihaljevic, Ruth Ladurner, Christian R. Beltzer

**Affiliations:** 1 Department of General, Visceral and Transplantation Surgery, University Hospital of Tübingen, Hoppe-Seyler-Straße 3, 72076 Tübingen, Germany; 2 Department of Surgery, Armed Forces Hospital, Ulm, Germany

**Keywords:** Acute appendicitis, Complicated appendicitis, Intraabdominal abscess, Antibiotic stewardship

## Abstract

Acute appendicitis is a common surgical emergency. Complicated appendicitis usually warrants perioperative antibiotic treatment in order to prevent infectious complications. Whether routine microbiological testing benefits the individual patient is a topic of debate. The goal of our study was to assess perioperative antibiotic prescriptions as well as the benefit of microbiological testing during the appendectomy as a predictor for bacteria encountered in infectious complications. This is a retrospective analysis of 1218 consecutive patients that underwent appendectomy at a tertiary referral center between 2014 and 2021. The patient charts were systematically analyzed regarding intraoperative outcome, microbiologic results, and postoperative infectious complications. 1218 patients were included in this study of which 768 were uncomplicated appendicitis (UA) and 450 were complicated appendicitis (CA). Microbiological testing was performed in 39.2% of UA cases (33.6% of which grew bacteria) compared to 74.9% of CA cases (78.6% positive cultures). The strongest individual predictors for SSI were gangrenous and perforated appendicitis. A total of 58 surgical-site infections developed, of which 49 were intra-abdominal fluid collections or abscesses. Thirty-two patients had revision surgery or CT-guided drainage for SSI. In the cases where microbiological testing was done both during the appendectomy and the SSI, 13/18 showed different bacteria on culture testing. The infectious outcome was favorable in 98.3%. While microbiological testing offers insights into resistance patterns, it is of little benefit for the individual patient, given the low predictive value for bacteria found during SSI. Achieving source control combined with empiric antibiotic coverage leads to favorable outcomes independent of culture results.

## Introduction

Acute appendicitis is the most common surgically treated pathology by emergency general surgery. Around 100 cases per 100,000 adults are diagnosed each year [1]. Even though there is increasing evidence that conservative treatment is an alternative to appendectomy in uncomplicated disease, surgery is still the gold standard of care [2]. While it is generally benign disease, complicated appendicitis (CA) can lead to sepsis and severe peritonitis with potential long-term consequences for the patient. In order to prevent postoperative infectious complications perioperative, antibiotics are generally administered [3]. While in uncomplicated appendicitis (UA), postoperative antibiotics can be safely omitted, and the optimal treatment duration for complicated appendicitis is yet to be determined [4–6]. The 2020 World Journal of Emergency Surgery (WSES) guidelines recommend a duration of 3–5 days of postoperative antibiotics depending on the patient condition and dynamic of recovery, but different durations have been described [5]. Even though appropriate antibiotic selection and duration should help prevent postoperative complications [3, 7], there is an ongoing debate whether antibiotic switch according to culture results provides a benefit for the patient [8–11]. This is further aggravated by the fact that final culture results, which are frequently poly-microbial, and antibiotic susceptibility testing are commonly only available several days after appendectomy. The topic remains important however since the rates of surgical-site infections (SSI) after appendectomy still vary between 0% and 11% [4]. Intra-abdominal abscesses (IAA), which might require interventional drainage or even surgical treatment, are reported with a rate of 6.4% following laparoscopic appendectomy, and as high as 24% in perforated appendicitis.

While the bacterial spectrum cultured from pus during appendectomies has been described repeatedly [12], there are limited data on the overlap of bacterial strains during appendectomy and subsequent SSI and IAA [13].

The aims of our study were to evaluate our perioperative antibiotic prescription strategy, the infectious outcome and the benefits of culture results for treating SSI.

## Methods

### Patient selection

This is a retrospective analysis of a consecutive cohort of adult patients (i.e., ≥ 16 years) who underwent appendectomy for acute appendicitis at our tertiary referral center between 2014 and 2021. Overall a total of 1218 patients were included in the final analysis.

### Data collection

All data were collected from the electronic patient record.

### Operative technique

Appendectomy was generally performed as a standardized three-port laparoscopic appendectomy with a 12 mm sub-umbilical, a 12 mm left lower quadrant port as well as a 5 mm suprapubic port. The meso-appendix was divided after thorough bipolar coagulation and, as needed, clip occlusion of a selectively identified appendiceal artery. The appendix base or the cecal pole was divided with a laparoscopic linear stapler. Open appendectomy was performed with a right lower quadrant incision. Conversion to open was done via a midline sub-umbilical laparotomy.

### Perioperative antibiotics

Perioperative antibiotics were administered at the surgeon’s discretion. Per protocol, the standard was cefotaxime 2000 mg and metronidazole 500 mg. Frequently used alternatives during the study period were ciprofloxacin 500 mg and piperacillin/tazobactam 4500 mg.

### Microbiological testing

Microbial cultures were also drawn at the operating surgeon’s discretion. The standardized technique was aspiration of pus/fluid and injecting this into a swab medium. Both anaerobes and aerobes were cultured. Strict anaerobes only underwent antibiotic resistance testing if they were found in monocultures. Aerobes and facultative anaerobes always underwent antibiotic resistance testing.

### Follow-up

There was no regular follow-up of patients in our outpatient department. Patients however were instructed to present to the surgical department in case of postoperative problems. Given that we are the only hospital providing emergency surgical care for our district, we work under the assumption that near all complications presented to our ER.

### Statistics

Statistical testing was performed with the Statistics Package for the Social Sciences (SPSS, IBM Version 28). Comparison between the patients with UA and CA was carried out using *Χ*^2^ test or Fisher’s exact test for nominal variables, and Mann–Whitney *U* test (MWU) for continuous variables, as appropriate. *p* Values of less than 0.05 were considered statistically significant. All *p* values are results of two-sided testing. Bonferroni correction was applied where necessary.

### Definitions

We defined complicated appendicitis when one or more of the following criteria were fulfilled: gangrenous or necrotizing appearance of the appendix, visible perforation of the appendix, presence of peritonitis with putrid fluid, and presence of an intra-abdominal abscess.

## Results

Overall 1218 patients were treated for acute appendicitis during the study period. According to the aforementioned criteria, 450 patients were classified as CA. Univariate analysis revealed equal sex distribution in both groups. Patients with CA were significantly older and had a higher BMI and ASA Score. They had a median symptom duration that was longer than in the UA group and were more likely to undergo preoperative CT scan. Procedures for CA were more likely to be converted to open and lasted significantly longer. These patients had a higher chance to undergo revision surgery, and postoperative length of stay was prolonged. For details, see Table 1.

**Table 1:** Baseline characteristics and perioperative findings

	Uncomplicated appendicitis (*n* = 768)	Complicated appendicitis (*n* = 450)	*p*
Sex male (%)	399 (52.0%)	247 (54.9%)	0.341
Age, mean in years (± SD)	32.3 (± 14.2)	48.6 (± 18.7)	< 0.001
Body mass index, mean in kg/m^2^ (± SD)	24.9 (± 5.0)	26.4 (± 5.1)	< 0.001
American Society of Anesthesia Score			
I	486 (63.2%)	164 (36.4%)	< 0.001
II	262 (34.1%)	227 (50.4%)	
III	20 (2.6%)	59 (13.1%)	
Duration of acute Symptoms, median in days (25–75%, IQR)	1 (1–2, IQR 1)	2 (1–3, IQR 2)	< 0.001
Preoperative diagnosis			
Ultrasound	751 (97.8%)	417 (92.7%)	< 0.001
Computed tomography	102 (13.3%)	226 (50.2%)	< 0.001
Operating time, median in min (25–75%, IQR)	47 (36–59, IQR 23)	63 (48–87, IQR 39)	< 0.001
Surgical strategy			
Laparoscopic appendectomy	763 (99.3%)	405 (90.0%)	< 0.001
Conversion to open appendectomy	1 (0.1%)	30 (6.7%)	
Open appendectomy	4 (0.5%)	15 (3.3%)	
Intraoperative findings			
(1) Gangrenous/necrotizing appendicitis		238 (52.9%)	
(2) Peri-appendicular abscess		145 (32.2%)	
(3) Perforated appendix		295 (65.6%)	
(4) Peritonitis		233 (51.8%)	
Any 1 criterion		175 (38.9%)	
Any 2 criteria		126 (28.0%)	
Any 3 criteria		111 (24.7%)	
Any 4 criteria		38 (8.4%)	
Length of stay median in days (25–75%, IQR)	2 (1–2, IQR 1)	3 (2–5, IQR 3)	< 0.001
Revision surgery within 90 days	9 (1.2%)	19 (4.2%)	< 0.001

Baseline characteristics and perioperative outcome

*SD* standard deviation; *IQR* Interquartile range

### Intraoperative outcomes

According to the surgical reports of the complicated cases, the appendix was classified as gangrenous in 52.9%, a peri-appendicular abscess was reported in 32.2%, the appendix appeared perforated in 65.6%, and peritonitis was present in 51.8%.

Any one criterion was fulfilled in 38.9% of CA while there was an overlap of two criteria in 28.0%, of three criteria in 24.7%, and of all four criteria in 8.4% respectively. Postoperative antibiotic continuation was significantly more common in CA patients at 72.2% compared to 9.9% in the UA group. For details, see Table 2.

**Table 2:** Infectious outcomes

	Uncomplicated appendicitis (*n* = 768)	Complicated appendicitis (*n* = 450)	*p*
Intraoperative cultures	301 (39.2%)	337 (74.9%)	< 0.001
No specimen sent for cultures	467 (60.8%)	113 (25.1%)	
Negative cultures	200 (66.4%)	72 (21.4%)	< 0.001
Positive cultures	101 (33.6%)	265 (78.6%)	
Continued antibiotics	76 (9.9%)	325 (72.2%)	< 0.001
Infectious complications	9 (1.2%)	49 (10.9%)	< 0.001
Superficial wound infection	4 (0.1%)	5 (1.1%)	< 0.001
Intra-abdominal wound infection/abscess	5 (0.1%)	44 (9.8%)	< 0.001

Infectious outcomes comparing the patients with UA to the ones with CA

### Postoperative antibiotics

We created a binary logistic regression model to explore predictors for continued antibiotic therapy after appendectomy in our cohort. The overall model was significant with a χ^2^ of 741.592, *p* < 0.001. The model accounted for approximately 45.8% (Cox and Snell *R*^2^) to 63.8% (Nagelkerke’s *R*^2^) of the variance and was able to correctly predict postoperative continued antibiotics in 87.4%. Hosmer and Lemeshow goodness-of-fit test indicated a decent fit as it was not significant *p* = 0.738. In the multivariate analysis ASA Score of 3, elevated WCC, high CRP, presence of gangrenous or perforated appendicitis, peritonitis, and microbiologic testing were independent predictors of prolonged antibiotics, where age and intraoperative finding of an abscess were not. The strongest predictors were visible perforation followed by high increased C-reactive protein (CRP), and the decision to send a microbiologic specimen. For details, see Table 3.

**Table 3:** Predictors for antibiotic continuation

Predictor	β	SE β	Wald’s χ^2^	df	*p*	Odds ratio	CI 95%
Constant	− 3.959	0.399	98.279	1	< 0.001	0.019	
Age	0.010	0.006	2.328	1	0.127	1.010	0.997–1.022
ASA I (ref)			8.535	2	0.014		
ASA II	0.215	0.214	1.008	1	0.315	1.240	0.815–1.886
ASA III	1.272	0.436	8.531	1	0.003	3.569	1.520–8.383
WCC	0.046	0.021	4.781	1	0.029	1.048	1.005–1.092
CRP	0.081	0.017	22.711	1	< 0.001	1.084	1.049–1.121
Gangrene	0.619	0.243	6.492	1	0.011	1.857	1.154–2.989
Abscess	0.401	0.374	1.146	1	0.284	1.493	0.717–3.111
Perforation	2.755	0.267	106.329	1	< 0.001	15.723	9.313–26.544
Peritonitis	0.849	0.244	12.073	1	< 0.001	2.337	1.448–3.772
Intraop. culture	0.867	0.192	20.291	1	< 0.001	2.380	1.632–3.470

Multivariate analysis of predictors for antibiotic continuation post-op

Cox and Snell *R*^2^ = 0.458. Nagelkerke *R*^2^ = 0.638. Accuracy 87.4%

*ASA* American Society of Anesthesiologists; *WCC* white cell count

Microbiological specimens were sent for culture in nearly 40% of UA and 75% of CA. Of the 301 cultures from UA cases, a third grew pathologic bacteria, while nearly four out of five cultures in the CA patients were positive. 65% of positive cultures were poly-microbial in the UA group compared to 89% poly-microbial positive cultures in the CA group. A total of 1224 bacteria were cultured with 147 different strains. The most common isolates were *E. coli* (*n* = 228, 18.7%) *Bacteroides fragilis* (*n* = 124, 10.1%), *Bacteroides thetaiotaomicron* (*n* = 94, 7.7%), *Bacteroides ovatus* (*n* = 60, 4.9%), *Bilophila wadsworthia* (*n* = 60, 4.9%), *Pseudomonas aeruginosa* (*n* = 57, 4.7%), *Streptococcus anginosus* (*n* = 44, 4.6%), *Enterococcus avium* (*n* = 36, 2.9%), *Enterococcus faecalis* (*n* = 28, 2.3%) and *Parabacteroides distasonis* (*n* = 28, 2.3%). There was no statistically significant difference between UA and CA although there was a trend toward more anaerobic bacteria in the CA group (50.1% vs 44.2%, *p* = 0.084).

### Surgical-site infections

Overall, there were 58 documented infectious complications in the form of surgical-site infections (4.8%), nine in the UA group—four superficial (0.5%) and five intra-abdominal SSI (0.6%) as well as 49 in the CA group—five superficial (1%) and 44 intra-abdominal SSI (9.8%).

In the CA group, of the four defining criteria, only gangrenous appendicitis (β 0.918, odds 2.505 (CI 95% 1.298–4.835), *p* = 0.006) and perforated appendicitis (β 1.006, odds 2.735 (CI 95% 1.180–6.338), *p* = 0.019) were independent risk factors for SSI, while the presence of peritonitis and abscess was not.

Of the nine patients with an SSI in the UA group six received antibiotics, compared to 48 of the 49 patients in the CA group (*p* < 0.001). In addition to antibiotics, no intervention was necessary in three UA patients (33.3%) compared to 16 CA patients (32.7%). Wound opening/superficial abscess drainage was performed in three UA patients (33.3%) compared to four CA patients (8.2%). CT-guided drains were placed in one UA patient (11.1%) vs 11 CA patients (22.4%). Revision surgery was performed in two UA patients (2.2%) vs 18 of CA patients (36.7%).

We compared the results of microbiological specimen collected from the initial surgery and during the SSI complication. Forty-three patients (74.1%) had material cultured during their appendectomy, whereas 15 (25.9%) did not. Thirty-seven patients (63.8%) had new microbiological testing done during the SSI.

Eighteen patients (31.0%) had either no material sent for culture or cultures yielded no growth. Twenty-two patients (37.9%) did not have microbiologic testing for their SSI. In the cases where bacteria were cultured during both the appendectomy and the SSI, all SSI bacteria were present on index swabs in five patients (8.6%), one different bacterium was cultured in six patients (10.3%), and more than one different/additional bacteria were grown in seven patients (12.1%). Therefore, 13 out of 18 patients (72.2%) that had both positive cultures during the appendectomy and the SSI had a different bacterial spectrum at the SSI that was not fully represented by the index swab.

In a next step, we examined whether antibiotics prescribed during SSI matched bacterial antibiotic resistances. Four patients (6.9%) did not receive any antibiotics. Seven patients (12.0%) either had no swab or showed no growth. The antimicrobial selection matched the bacteria encountered during the appendectomy and the SSI in twelve cases (20.7%). In 18 cases (31.0%), it matched only the index appendectomy but not the SSI, (nine patients had no swab during the SSI and six grew no bacteria). The remaining three grew bacteria that were not covered by the chosen antibiotic regimen. In eight cases (13.8%), the empiric antimicrobial during the SSI matched the bacteria cultured from the SSI but not the index bacteria (three cases grew bacteria, that were not properly covered, no initial cultures were done in five). In nine patients (15.5%), even though there were microbiologic results available from the appendectomy and the SSI, the antibiotics did not fully cover either.

With the exception of four patients who had superficial wound infections opened in the emergency room, all patients had antibiotic treatment.

Overall, the outcome was favorable in 57 out of 58 patients with the remaining patient requiring further intervention due to an anastomotic leak after initial appendectomy followed by a leak of the appendix base treated with ileocecal resection.

## Discussion

Acute appendicitis remains the most commonly treated disease in acute care surgery. The lifetime risk of developing appendicitis is around 8% in Europe and 9% in the US [14]. While conservative treatment with antibiotics and anti-inflammatory agents may be sufficient for uncomplicated disease, surgical removal of the appendix is the gold standard to date. In high resource settings, laparoscopic appendectomy is now the standard approach [15] and over 95% of appendectomies are performed that way, while less than 10 years ago, open appendectomy was still the standard of care in many European countries [12].

For most patients, source control through appendectomy with a perioperative antibiotic single shot antibiotic is sufficient therapy of acute appendicitis. Neither the WSES nor the European Association for Endoscopic Surgery (EAES) guidelines recommend a specific antibiotic combination as the standard perioperative regimen for adults but rather recommend the perioperative single shot to be in accordance to the local bacteria and their resistance pattern [4, 5]. The treatment duration in complicated appendicitis has been studied in various trials and analyses [16, 17] and is currently recommended to be no longer than 3–5 days [5, 18], while for cases of uncomplicated appendicitis, postoperative antibiotics are generally not recommended. We were surprised to find nearly 10% of patients in the UA group to receive additional postoperative antibiotics and there is no easy explanation for it. Our regression model showed that surgeons in our team were more eager to continue antibiotics post appendectomy when specimens were sent during appendectomy and when patients were having more severe comorbidities (as suggested by the higher ASA-Scores). Cloudy fluid encountered during appendectomy might have also led to surgeons over-staging the severity of disease.

### Value of culture results

While our cohort goes back nearly 10 years, and some recommendations may have changed in the meantime, it is fairly safe to say that routine microbiological testing is unnecessary given the low benefit for the patient at the expanse of an increased cost.

The value of microbiological cultures and resistance testing lies in the information it provides for the local antibiotic stewardship teams and guideline creators, more than for the individual patient. Culture results offer insights into prevalence of multi-resistant Gram-negative bacteria (MRGN) or Gram-positive bacteria (e.g., vancomycin-resistant *Enterococcus*). This of course is influenced by the overall prevalence of resistant bacteria in the local population [19]. The high incidence of appendicitis and the variety of surgeons treating the disease call for standardization of diagnostics, treatment regimen and duration. Antimicrobial stewardship has only begun to intervene in the field of intra-abdominal infections [20]. While most studies focus on the reduction of antibiotic use, there are also studies highlighting the benefit of intraoperative cultures and a strict postoperative antibiotic regimen introduced following local microbiological findings of the recent past in order to lower SSI [21, 22]. The general advice to de-escalate antibiotics after resistance testing is done is rarely followed for abdominal infections. Most abdominal infections are poly-microbial, need interventional or surgical source control and by the time proper resistance testing is done, the antibiotics can safely be stopped [23].

We advocate for center-specific antibiotic recommendations, whenever possible, according to microbiological culture results specific for the disease, for which the recommendations are made, since there may be vast differences between antibiotic resistance patterns of bacteria grown in the general population and a targeted subpopulation, as has been described by our group for urinary tract infections in kidney transplant recipients [24].

In the wake of the exploration of the human microbiome, the appendiceal microbiome has also been studied and shown to be different between uncomplicated and complicated appendicitis [25]. Similarly, several groups have described different bacterial spectrums between UA and CA or perforated and unperforated cases [26, 27]. This is a finding that we can hardly reproduce. In our study, a total of 1224 bacterial strains were cultured with about 4-to-1 ratio in the CA group. While there was a tendency toward more anaerobic bacteria in the complicated cases, the distribution of the more pathogenic or complex-to-treat bacteria, such as *E. coli*, *Klebsiella*, *Pseudomonas*, and *Enterococcus* species, was done in a near-identical ratio. Coccolini and colleagues tried to describe bacteria based on a multi-centric multi-continental study. In their study, 704 bacteria were isolated from 1431 patients (of which more than half had open appendectomy) [12]. They reported only 11% of bacteria to be strict anaerobes, which is different from our cohort where nearly 50% of isolated bacteria were anaerobes. Thus, we conclude that bacterial culture results are not only dependent on the culture processing but also the method of specimen acquisition which may be affected by the surgical approach. Given the high rate of anaerobes, appropriate beta-lactams or combination therapy of a cephalosporin and metronidazole should be the standard antibiotic regimen. While we did see an increase in piperacillin-resistant *Bacteroides* strains, the addition of metronidazole to piperacillin–tazobactam did not seem to alter the already favorable outcomes. Shang et al. retrospectively compared the outcomes of pediatric patients that either received a broad-spectrum beta-lactam with metronidazole to those that did not receive metronidazole and did not see a difference in infectious complications or overall outcome [28].

So how often should antibiotic guidelines be updated? The only study to our knowledge to explore antimicrobial resistance over a 20-year period found no increase in resistant bacteria in pediatric appendicitis patients in France [29]. Whether these results can be safely adopted for adults is open for debate. We recently published a matched pair analysis that found the antibiotic resistance patterns to be near-identical between young adult and elderly patients, which may hint in the same direction [30].

### Comparison of culture results during appendectomy and infectious complications

While it would appear logical that intra-abdominal or subcutaneous abscesses develop in patients that were treated by an insufficient selection or duration of postoperative antibiotic [7], data show that postoperative antibiotic switch according to culture results has limited influence on abscess formation [8–11]. Pena et al. recently published a cohort of 270 patients with complicated appendicitis split in two groups of which one had cultures drawn during the appendectomy with subsequent antibiotic switch based on culture results and a total treatment time of 7 days in both groups. Patients who underwent antibiotic switch developed IAA in 21.1% compared to 13.3% in their complicated cases overall [11]. This was not randomized trial however and may be slightly biased by more severe clinical features in the first group, as suggested by the authors. Further, more severe cases are less likely to improve clinically and thus warrant antibiotic switches.

There are increasing data that bacteria encountered during SSI after appendectomy are different from the ones cultured during the initial procedure. Foo et al. described only an overlap of 31% between their intraoperative and SSI cultures [10]. Similar numbers were recently published by Dahlberg et al. who found a 29% overlap in their cohort of pediatric IAA patients [13]. In our study, we found nearly identical results. We found a mismatch of bacteria in 72.2% and respectively only 27.8% of patients with positive cultures during appendectomy and SSI did not grow additional bacteria during the infectious episode. Even though the overlap was low and the antibiotics chosen during SSIs frequently did not cover all bacteria, the outcome was favorable in 98.3% of cases.

### Strength and limitations

This a retrospective trial comparing the outcome of UA and CA in over 1200 patients over a 7-year time period. The definition of CA was made purely on the reported characteristics in the OP note. There were no intraoperative photos available to the authors; therefore, no secondary assessment was available.

## Conclusion

Here we present a cohort of 1218 consecutive patients treated for appendicitis at a tertiary medical center. While microbiological testing offered insights into resistance patterns, it is of little benefit for the individual patient, given the low predictive value for bacteria found during surgical-site infections. Achieving source control combined with empiric antibiotic coverage leads to favorable outcomes independent of culture results, which suggests that cultures can safely be omitted.

## Data Availability

Anonymized data can be provided on further request.
